# Effects of IL-4-590C/T (rs2243250) Polymorphism on the Susceptibility of Smoking-Related Cancer: A Meta-Analysis Involving 11,407 Subjects

**DOI:** 10.1155/2019/3104176

**Published:** 2019-12-02

**Authors:** Guangyuan Chen, Cong Hu, Yuxuan Song, Haifei Zhang, Song Li, Penghui Lai, Yiling Zhang, Peng Huang

**Affiliations:** ^1^The Second Clinical Medical School, Nanchang University, Nanchang, Jiangxi 330006, China; ^2^Department of Urology, Tianjin Medical University General Hospital, Tianjin 300052, China; ^3^Center for Evidence-Based Medicine, School of Public Health, Nanchang University, Nanchang 330006, China; ^4^Jiangxi Province Key Laboratory of Preventive Medicine, School of Public Health, Nanchang University, Nanchang 330006, China

## Abstract

**Background:**

Several previous studies have assessed the relationship between IL-4-590C/T gene polymorphism and smoking-related cancer in recent years; however, the results remain controversial. Based on it, the study intends to clarify whether IL-4-590C/T variant increases the risk of smoking-related cancer through meta-analysis.

**Methods:**

We searched PubMed, EMBASE, Web of Science, Cochrane Library database, China National Knowledge Infrastructure, and Wanfang data information service platform to collect qualified case-control studies in strict accordance with the inclusion and exclusion standards. The 95% confidence interval (95% CI) and its odds ratio (OR) were adopted to access the relation between IL-4-590C/T gene polymorphism and smoking-related cancer; sensitivity analysis and publication bias assessment were carried out after the studies' quality evaluation.

**Results:**

17 studies were included in total, with 5,061 patients and 6,346 control cases. A significant association between IL-4-590C/T variant and smoking-related cancer in total population was revealed in our meta-analysis results, and IL-4-590C/T variant might have a relatively protective effect on smoking-related cancer (CT vs. TT: *P*=0.026, OR = 0.900, 95% CI: 0.820–0.987). Subgroup analysis by ethnicity showed that the IL-4-590C/T polymorphism was associated with a decreased risk of smoking-related cancer in the Asian population (CT vs. TT: *P*=0.008, OR = 0.878, 95% CI: 0.798–0.967; CC + CT vs. TT: *P*=0.030, OR = 0.903, 95% CI: 0.824–0.990). Subgroup analysis based on types of cancer demonstrated the IL-4-590C/T variant achieved a lower risk in renal cell cancer (CC vs. TT: *P*=0.046, OR = 0.640, 95% CI: 0.412–0.993).

**Conclusion:**

There is a conspicuous association between IL-4-590C/T polymorphism and decreased risk of smoking-related cancer, particularly in Asians. And IL-4-590C/T polymorphism may have a protective effect on renal cell cancer.

## 1. Introduction

Smoking is currently an important risk factor for a variety of cancers. Almost half of male urinary tumors and 1/3 of female urinary tumors are caused by smoking [1]. Compared with nonsmokers, smokers can increase the prevalence of bladder cancer by two to three times [2]. Cigarette smoke itself is a rich free radical that induces DNA damage in cells by inducing it as a triggering factor for tumors. In addition, more than 60 carcinogens have been found in cigarette smoke, like aromatic amines, polycyclic aromatic hydrocarbons, and specific nitrosamines in tobacco; all of them are recognized as carcinogens in humans [3–5]. The genotoxic patterns of these chemicals have been delineated and related to the DNA damage in cells. DNA adducts can be caused by smoking [6], as a result of the formation of covalently bound DNA damage by the production of electrophilic substances. The formation of DNA adducts is a cancerous potential result, and DNA adducts can mislead DNA replication, resulting in mutations [7]. These specific genetic mutations affect critical areas that control cell function, which can lead to tumorigenesis [8]. Compared with individuals who smoke slightly, individuals who smoke heavily have a higher risk of cancer because of the higher concentration of carcinogens in their body [9, 10]. The International Agency for Research on Cancer (IARC) has defined smoking-related cancer as the cause of cancers of the lung, oral cavity, gastric, bladder, and so on [11]. According to the latest cancer report of 2018, smoking-related cancers became a major public health problem with extremely high morbidity and mortality [12].

Interleukin-4 (IL-4) gene is oriented in the cytokine gene cluster on chromosome 5q31-33. It is a cytokine that can promote the proliferation of Th2 cells while inhibit the proliferation of Th1 cells, and finally reduce the immune response mediated by Th1 [13]. Many studies have shown that interleukin-4 can promote tumor progression and metastasis by affecting apoptosis of tumor cells [14–17]. At present, there have been several case-control researches to explore the relationship between IL-4-590C/T (rs2243250) gene polymorphism and smoking-related cancer, but the results are still controversial. The polymorphism of IL-4-590C/T (rs2243250) is a C to T base mutation, and the T-allele gene means mutational allele gene and C-allele gene means the wild allele gene. Our aim is to study whether the mutational T-allele and TT genotype increase the susceptibility of smoking-related cancer compared with CT and CC containing the wild C allele through systematic review and meta-analysis.

## 2. Methods

### 2.1. Literature Retrieval

We performed a comprehensive search of PubMed, EMBASE, Web of Science, Cochrane Library database, China National Knowledge Infrastructure, Wanfang data information Service platform, and manual search to find relevant studies. The English search strategy: (cancer or carcinoma or neoplasm) AND (IL 4 OR IL-4 OR Interleukin 4 OR Interleukin-4) AND (SNP or variant or polymorphism or genotype). We searched the studies published before Aug 1, 2019. At the same time, the corresponding literature, academic conference papers, and unpublished documents were included in the literature through manual search.

### 2.2. Inclusion and Exclusion Criteria

#### 2.2.1. Inclusion Criteria

The inclusion criteria were as follows: (1) case-control articles about the polymorphism of IL-4 referring to smoking-related cancers; (2) the studies including IL-4-590C/T (rs2243250) variant; (3) studies having sufficient data for examining the odds ratio (OR) with 95% confidence intervals (CIs); (4) the articles reporting the risk of smoking-related cancers which were defined according to the IARC monograph; (5) the genotypes accorded with Hardy–Weinberg equilibrium (HWE).

#### 2.2.2. Exclusion Criteria

The exclusion criteria were as follows: (1) the control group gene distribution contained in these studies was not subject to HWE; (2) literature with incomplete data or data not available.

### 2.3. Literature Quality Evaluation and Data Extraction

The quality of the included literature was accessed using the evaluation criteria of the case-control study in the Newcastle–Ottawa scale (NOS). The evaluation included (1) the selection of study subjects, (2) the inter-group comparability, and (3) the exposure evaluation. The NOS divides the quality of the literature into 0–9 points. The higher the score, the better the quality of the literature. The score ≥5 is considered to be a literature of high quality.

The data extraction includes the studies' names, the publication year, the target country, the race, the cancer type, the amount of case group and control group, and its genotype distribution and genotyping methods.

### 2.4. Statistical Methods

Meta-analysis was performed using Stata12.0 software. *Q* test and heterogeneity coefficient *I*^2^ were used to judge the heterogeneity between studies. If there is statistical heterogeneity (*I*^2^ > 50%, or *P* < 0.1), the random effect model is used for meta-analysis; otherwise, the fixed-effect model is used. Using odds ratio (OR) as an effector to reflect the strength of the correlation between IL-4 rs2243250 and smoking-related cancer. And we included five models: (1) allele model, (2) dominant model, (3) codominant model, (4) homozygote model, and (5) recessive model, to analyze the association, respectively. The T allele is a mutational allele, and the C allele is a wild allele. In addition, the heterogeneity source was explored by subgroup analysis. And the *P* value of Egger's test, Begg's test, and funnel chart was obtained to evaluate the bias induced from publication.

## 3. Results

**Figure 1:**
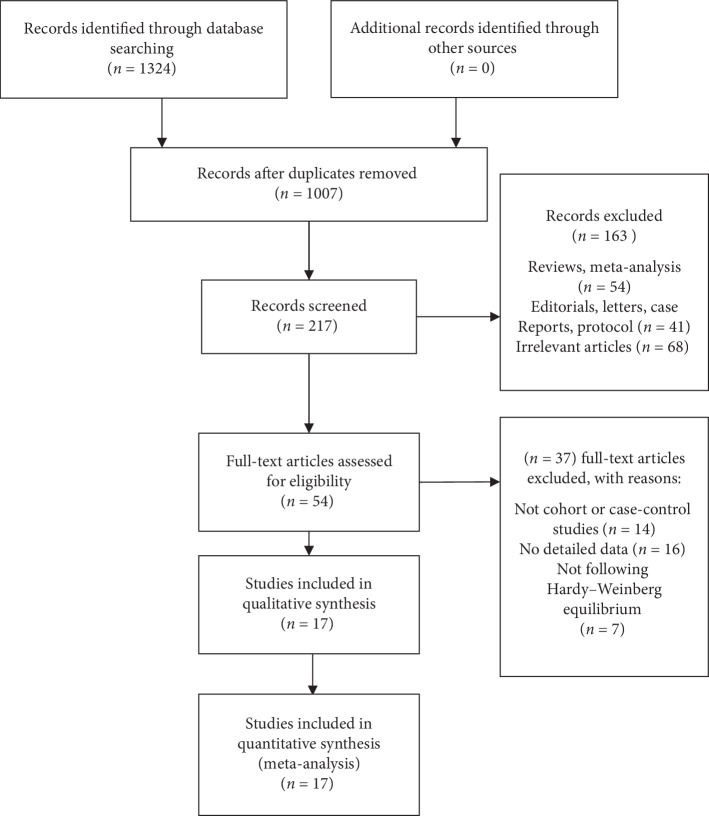
Flowchart illustrating the search strategy for IL-4-590C/T polymorphism and the risk of smoking-related cancer.

A total of 1,324 potentially relevant researches were identified in accordance with the search strategy (Figure 1). Ultimately, 17 case-control articles were selected into our meta-analysis.

### 3.1. Traits of the Selected Studies

The traits of the selected studies are presented in Table 1. For IL-4 rs2243250 polymorphism, 24 articles were investigated. However, deviations from HWE were found in 7 articles [18–24], so we have to remove these 7 articles (Figure 1), while the other 17 articles were in accordance with HWE. Of these 17 articles [25–41] (including 5,061 cases and 6,346 controls), seven studied the relation between IL-4-590C/T variant and the susceptibility of gastric cancer, three between IL-4-590C/T variant and the susceptibility of lung cancer, and three between IL-4-590C/T variant and the susceptibility of oral carcinoma. The others studied the association between studied IL-4-590C/T variant and the susceptibility of bladder cancer, hepatocellular cancer, and renal cell carcinoma. For genotyping methods, seven were using PCR-RFLP, six were using TaqMan, three were using PCR, and one was using MASSARRAY. The NOS scores of the 17 documents were all more than 5, meaning that all of them were high-quality studies (Table 1).

### 3.2. Meta-Analysis Results

Table 2 listed the main results of the meta-analysis of IL-4-590C/T (rs2243250) gene polymorphism and the risk of smoking-related cancer in overall population.  Table 3 shows the main results of subgroup analysis by ethnicity.

#### 3.2.1. Association between IL-4-590C/T Polymorphism and the Risk of Smoking-Related Cancer

**Figure 2:**
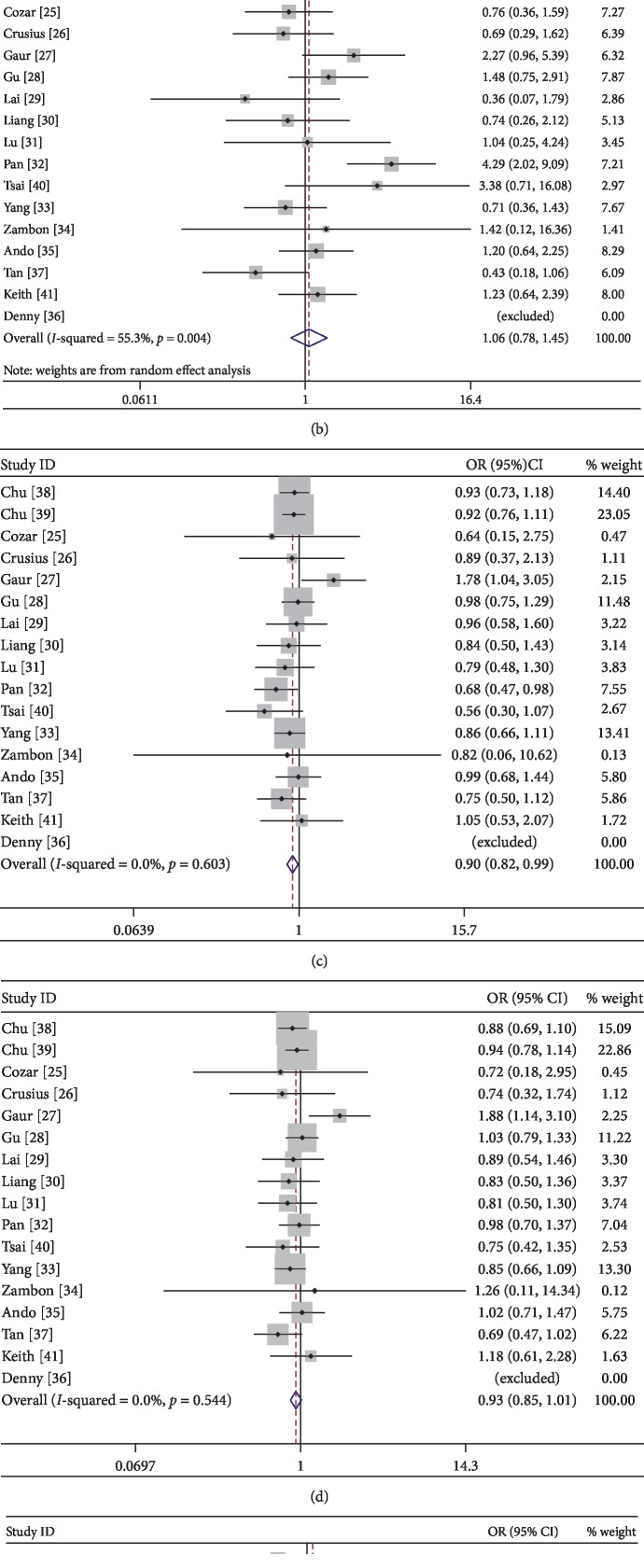
Forest plot of association between IL-4-590C/T polymorphism and smoking-related cancer in overall population ((a) C allele vs. T allele; (b) CC vs. TT; (c) CT vs. TT; (d) CC + CT vs. TT; (e) CC vs. CT + TT).

Seventeen articles including 5,061 cancer cases and 6,346 normal controls were investigated. As illustrated in Table 2, a significant relation was found for IL-4-590C/T gene polymorphism and the risk of smoking-related cancer in overall population (CT vs. TT: *P*=0.026, OR = 0.900, 95% CI: 0.820–0.987). And the CT genotype carriers have a slightly lower incidence of smoking-related cancer compared to that of TT carriers and the TT variants might be a potential risk factor for smoking-related cancer susceptibility in overall populations (Table 2 and Figure 2).

#### 3.2.2. Association between IL-4-590C/T Polymorphism and Smoking-Related Cancer Risk in Subgroup Analysis by Ethnicity

The heterogeneity of IL-4-590C/T variant and smoking-related cancer was complicated by multiple indexes, so subgroup analysis of different ethnicities was carried out. A significant association between IL-4-590C/T variant and the susceptibility of the smoking-related cancer in Asian population (CT vs. TT: *P*=0.008, OR = 0.878, 95% CI: 0.798–0.967; CC + CT vs. TT: *P*=0.030, OR = 0.903, 95% CI: 0.824–0.990) was found. Our results indicated that CC/CT genotype carriers had a lower risk compared with TT carriers and the CC/CT variant might be a protective factor for smoking-related cancer susceptibility in Asian population. However, any association between smoking-related cancer risk and IL-4-590C/T variant was found in Caucasians (Table 3).

#### 3.2.3. Association between Smoking-Related Cancer Susceptibility and IL-4-590C/T Polymorphism in Subgroup Analysis by Cancer Type

**Figure 3:**
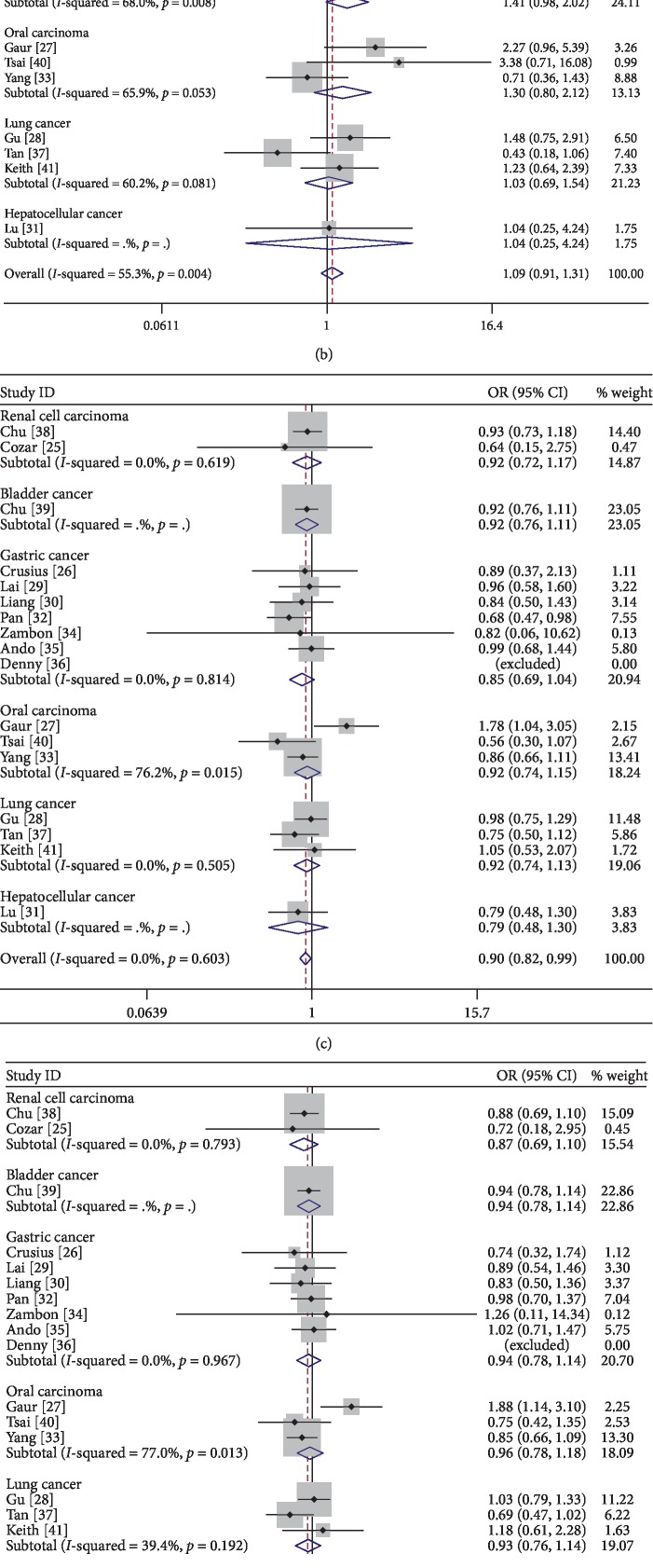
Forest plot of subgroup analysis by cancer type ((a) C allele vs. T allele; (b) CC vs. TT; (c) CT vs. TT; (d) CC + CT VS. TT; (e) CC vs. CT + TT).

Our results demonstrated that IL-4-590C/T polymorphism had a lower risk for renal cell cancer (CC vs. TT: *P*=0.046, OR = 0.640, 95% CI: 0.412–0.993). And the CC genotype might be a protective factor in renal cell cancer. However, for bladder cancer, gastric cancer, oral carcinoma, lung cancer, and hepatocellular cancer, we did not find any obvious association in different genotype carriers (Figure 3).

### 3.3. Sensitivity Analysis

**Figure 4:**
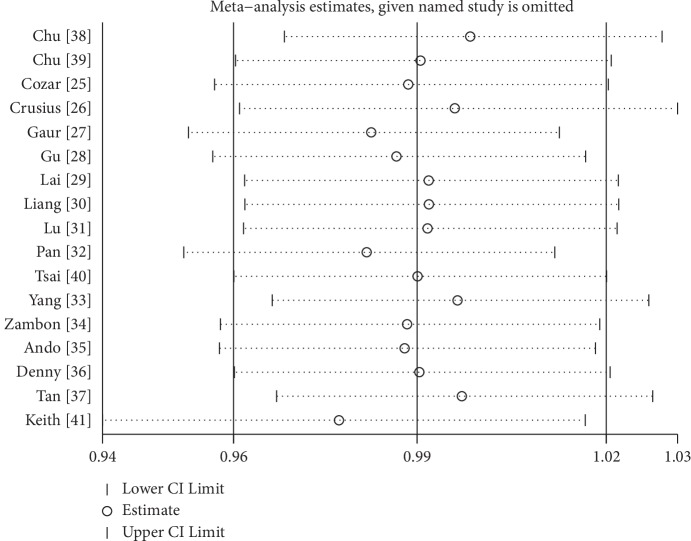
Sensitivity analysis of the pooled ORs and 95% CIs for IL-4-590C/T polymorphism (C allele vs. T allele).

Sensitivity analysis was performed by deleting a study one by one, and the combined results showed no significant changes, denoting that the results of this study were relatively steady (Figure 4).

### 3.4. Publication Bias

**Figure 5:**
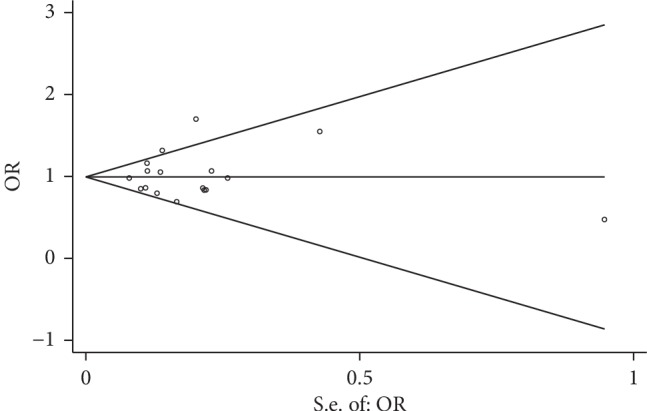
Begg's funnel plot with pseudo 95% confidence limits for studies of the association between smoking-related cancer risk and IL-4-590C/T polymorphism (C allele vs. T allele).

Publication bias was evaluated by Begg's funnel plots and Egger's test (the allelic contrast of pooled analysis: Egger's test, *P*=0.656; Begg's test, *P*=0.592), and the *P* value of Egger's test and Begg's test were all more than 0.05, suggesting that there was no publication bias for the association between IL-4 rs2243250 variant and the susceptibility of smoking-related cancer in these included studies (Table 2 and Figure 5).

## 4. Discussion

In terms of originality, our meta-analysis is the first paper to study the relationship between IL-4-590C/T (rs2243250) polymorphism and smoking-related cancer. We use five genetic models ((1) allele model; (2) dominant model; (3) codominant model; (4) homozygote model; (5) recessive model) to determine the association between IL-4-590C/T (rs2243250) polymorphism and smoking-related cancer. As a result, we found that IL-4-590C/T (rs2243250) polymorphism was associated with the decreased risk of smoking-related cancer in overall population. A slightly lower incidence of smoking-related cancer was observed in CT carriers compared to TT carriers and the TT variant might be a risk factor resulting in smoking-related cancer susceptibility in different races.

In subgroup analysis by ethnicity, our results indicated that the polymorphism of IL-4-590C/T was associated with the decreased risk of smoking-related tumors in Asian population. The CT/CC genotype was a protective factor for the susceptibility of smoking-related cancer while the TT genotype was a risk factor for smoking-related cancer susceptibility. No obvious association was observed in the population of Caucasians. It might account for that genetic polymorphisms are greatly different in various racial groups, which means that we can pay more attention to IL-4-590C/T gene polymorphism when screening for smoking-related cancer, especially among Asians.

For cancer-type subgroup analysis, the susceptibility of renal cell carcinoma was observed to be associated with the polymorphism of IL-4-590C/T. And the CC genotype might be a protective factor in renal cell cancer.

Our results were consistent with Chu et al. [38]; they found that the polymorphism of IL-4 rs2243250 was also associated with the lower risk of renal cell carcinoma. But our result was different from Cozar et al. [25]; they did not find that the polymorphism of IL-4 rs2243250 was also associated with the susceptibility of renal cell carcinoma. In addition, Pan et al. [32] and Tan et al. [37] have found that IL-4-590C/T variant was relevant with the increased risk of lung cancer and gastric cancer. The possible reasons for the different results of the above studies have been summarized as follows: (1) the genetic background of the population was different; (2) the sample volume of Asians we included in this meta-analysis was much larger than that of Caucasians; (3) the methods of genotyping were different in these included studies; (4) the different types of smoking-related cancers had different relationships with IL-4 rs2243250 polymorphism. In addition, IL-4 rs2243250 polymorphism was involved in the metabolism of a variety of carcinogens associated with tobacco smoke, which may be affected by complex factors like multiple genes, environmental factors, individual genetic background, and dietary habits.

Some advantages feature our findings. First, we performed the NOS quality test on all the included literature, ensuring that the literature works included were of high quality. Second, HWE tests were conducted on included documents, and we excluded those that did not meet the Hardy–Weinberg Equilibrium. Third, more than 11,407 subjects consisting of 5,061 patients with smoking-related cancer and 6,346 controls were contained in the study. Therefore, the sample size was large enough to confirm the results of our analysis. Moreover, sensitivity analysis and Egger's test showed the results were stable.

One of the important things about meta-analysis is heterogeneity, and ignoring the heterogeneity may lead to scientific errors. We found the heterogeneity was significant in many models. Therefore, we conducted a subgroup analysis and finally found that the type of cancer may be the source of this heterogeneity.

There are still some limitations in this study that cannot be avoided. Firstly, some of the included studies show lack of enough original information like smoking history and family conditions. So it cannot be assessed for potential interactions with interference factors. Besides, the present meta-analysis only included English and Chinese studies, so it might cause language bias. Therefore, there is a need for more multicentric researches with large samples to be carried out in future to gain more insights into the association between IL-4 rs2243250 polymorphism and smoking-related cancer.

## 5. Conclusion

Our study indicates that smoking-related cancer susceptibility is associated with IL-4-590C/T polymorphism in the total population. The CT genotype carriers have a slightly lower incidence of smoking-related cancer compared to TT carriers in the overall populations. And the IL-4-590C/T polymorphism has different susceptibility to smoking-related tumors in the different populations, especially in Asians. However, in order to determine a comprehensive conclusion on the correlation between smoking-related cancer susceptibility and IL-4-590C/T polymorphism, more prospective cohort studies are still needed in the future.

## Figures and Tables

**Table 1:** Main characters of studies included in this meta-analysis.

Author	Year	Country	Ethnicity	Cancer type	Genotyping method	Case/*n*	Control/*n*	*P* for HWE	NOS scores
Amirzargar et al. [18]	2005	Iran	Caucasian	Leukemia	PCR-SSP	30	40	*P* < 0.05	6
Chang et al. [19]	2015	China	Asian	Lung cancer	PCR-RFLP	358	716	*P* < 0.05	8
Chu et al. [39]	2012	China	Asian	Bladder cancer	TaqMan	816	1140	0.323	7
Chu et al. [38]	2012	China	Asian	Renal cell carcinoma	TaqMan	620	623	0.079	7
Crusius et al. [26]	2008	Netherlands	Caucasian	Gastric cancer	PCR-RFLP	242	1154	0.603	7
El-Omar et al.[20]	2003	Scotland	Mixed	Esophageal cancer	PCR-ARMS	90	209	*P* < 0.05	6
El-Omar et al. [20]	2003	Scotland	Mixed	Gastric cancer	PCR-ARMS	122	209	*P* < 0.05	7
Gaur et al. [27]	2011	India	Caucasian	Oral carcinoma	PCR-RFLP	140	120	0.095	8
Gu and Shen [28]	2014	China	Asian	Lung cancer	TaqMan	500	500	0.348	7
Lai et al. [29]	2005	China	Asian	Gastric cancer	PCR-RFLP	123	162	0.698	7
Li et al. [21]	2012	China	Asian	Lung cancer	PCR-RFLP	1072	1126	*P* < 0.05	7
Liang et al. [30]	2010	China	Asian	Gastric cancer	PCR	238	112	0.118	7
Lu et al. [31]	2014	China	Asian	Hepatocellular cancer	PCR	154	170	0.549	7
Olson et al. [22]	2007	USA	Mixed	Prostate cancer	PCR-RFLP	149	128	*P* < 0.05	8
Pan et al. [32]	2014	China	Asian	Gastric cancer	PCR	308	307	0.389	7
Saxena et al. [23]	2014	India	Caucasian	Hepatocellular cancer	TaqMan	59	153	*P* < 0.05	7
Tsai et al. [40]	2005	China	Asian	Oral carcinoma	PCR-RFLP	130	105	0.741	7
Yang et al. [33]	2014	China	Asian	Oral carcinoma	PCR-RFLP	463	623	0.233	7
Zambon et al. [34]	2008	Italy	Caucasian	Gastric cancer	TaqMan	40	64	0.800	7
Ando et al. [35]	2009	Japan	Asian	Gastric cancer	TaqMan	330	190	0.248	7
Kesarwani et al. [24]	2008	India	Asian	Prostate cancer	PCR-ARMS	200	200	*P* < 0.05	7
Denny et al. [36]	2018	Spanish	Caucasian	Gastric cancer	PCR-RFLP	15	20	0.814	8
Tan et al. [37]	2019	China	Asian	Lung cancer	MASSARRAY	199	266	0.195	7
Keith et al. [41]	2018	USA	Caucasian	Lung cancer	TaqMan	616	616	0.338	9
Cozar et al. [25]	2007	Spain	Caucasian	Renal cell carcinoma	RCR-RFLP	127	174	0.844	7

*P* for HWE: *P* value for Hardy–Weinberg equilibrium; PCR: polymerase chain reaction; RFLP: restriction fragment length polymorphism; ARMS: amplification refractory mutation system; SSP: sequence specific primers.

**Table 2:** Meta-analysis results.

Contrast model	OR (95% CI)	*P*	Test for heterogeneity		Publication bias (Egger's test)		Publication bias (Begg's test)		Analysis model
			*I* ^2^ (%)	*P*	*t*	*P*	*Z*	*P*	
C vs. T	0.981 (0.887, 1.084)	0.700	44.60	0.025	0.45	0.656	0.54	0.592	R
CC vs. TT	1.065 (0.783, 1.448)	0.689	55.30	0.004	0.35	0.733	0.68	0.499	R
CT vs. TT	0.900 (0.820, 0.987)	0.026	0.00	0.603	0.02	0.984	0.05	0.964	F
CC + CT vs. TT	0.928 (0.849, 1.014)	0.098	0.00	0.544	0.37	0.720	0.77	0.444	F
CC vs. TT + CT	1.104 (0.860, 1.416)	0.438	59.20	0.001	0.97	0.348	0.70	0.484	R

R: random effect model; F: fixed-effect model; OR: odds ratio; CI: confidence interval.

**Table 3:** Subgroup meta-analysis by ethnicity of IL-4 rs2243250.

Subgroup	Contrast model	OR (95% CI)	*P*	Test for heterogeneity		Analysis model
				*I* ^2^ (%)	*P*	
Asian	C vs. T	0.947 (0.877, 1.023)	0.168	28.40	0.175	F
	CC vs. TT	1.047 (0.698, 1.571)	0.825	64.90	0.001	R
	CT vs. TT	0.878 (0.798, 0.967)	0.008	0.00	0.806	F
	CC + CT vs. TT	0.903 (0.824, 0.990)	0.030	0.00	0.909	F
	CC vs. TT + CT	1.097 (0.726, 1.658)	0.659	67.10	0.001	R

Caucasian	C vs. T	1.124 (0.858, 1.471)	0.397	59.90	0.029	R
	CC vs. TT	1.113 (0.763, 1.624)	0.580	19.70	0.289	F
	CT vs. TT	1.259 (0.875, 1.813)	0.215	0.00	0.487	F
	CC + CT vs. TT	1.341 (0.948, 1.897)	0.097	11.40	0.341	F
	CC vs. TT + CT	1.046 (0.882, 1.241)	0.605	42.70	0.121	F

R: random effect model; F: fixed-effect model; OR: odds ratio; CI: confidence interval.

## Abbreviations

OR: Odds ratio
CI: Confidence interval
HWE: Hardy–Weinberg equilibrium
IL-4: Interleukin-4
SNP: Single nucleotide polymorphism
IARC: International Agency for Research on Cancer
The NOS scale: The Newcastle–Ottawa scale
R: Random effect model
F: Fixed-effect model.

## Supplementary Materials

Supplementary Materials PRISMA 2009 Checklist.
